# Cardiac Expression of Microsomal Triglyceride Transfer Protein Is Increased in Obesity and Serves to Attenuate Cardiac Triglyceride Accumulation

**DOI:** 10.1371/journal.pone.0005300

**Published:** 2009-04-23

**Authors:** Emil D. Bartels, Jan M. Nielsen, Lars I. Hellgren, Thorkil Ploug, Lars B. Nielsen

**Affiliations:** 1 Department of Clinical Biochemistry, Copenhagen University Hospital Rigshospitalet, Copenhagen, Denmark; 2 Department of Cardiology, Aarhus University Hospital, Skejby, Denmark; 3 Department of Systems Biology and Centre for Advanced Food Studies, Technical University of Denmark, Lyngby, Denmark; 4 Department of Biomedical Sciences, University of Copenhagen, Copenhagen, Denmark; University of Camerino, Italy

## Abstract

Obesity causes lipid accumulation in the heart and may lead to lipotoxic heart disease. Traditionally, the size of the cardiac triglyceride pool is thought to reflect the balance between uptake and β-oxidation of fatty acids. However, triglycerides can also be exported from cardiomyocytes via secretion of apolipoproteinB-containing (apoB) lipoproteins. Lipoprotein formation depends on expression of microsomal triglyceride transfer protein (MTP); the mouse expresses two isoforms of MTP, A and B. Since many aspects of the link between obesity-induced cardiac disease and cardiac lipid metabolism remain unknown, we investigated how cardiac lipoprotein synthesis affects cardiac expression of triglyceride metabolism-controlling genes, insulin sensitivity, and function in obese mice. Heart-specific ablation of MTP-A in mice using Cre-loxP technology impaired upregulation of MTP expression in response to increased fatty acid availability during fasting and fat feeding. This resulted in cardiac triglyceride accumulation but unaffected cardiac insulin-stimulated glucose uptake. Long-term fat-feeding of male C57Bl/6 mice increased cardiac triglycerides, induced cardiac expression of triglyceride metabolism-controlling genes and attenuated heart function. Abolishing cardiac triglyceride accumulation in fat-fed mice by overexpression of an apoB transgene in the heart prevented the induction of triglyceride metabolism-controlling genes and improved heart function. The results suggest that in obesity, the physiological increase of cardiac MTP expression serves to attenuate cardiac triglyceride accumulation albeit without major effects on cardiac insulin sensitivity. Nevertheless, the data suggest that genetically increased lipoprotein secretion prevents development of obesity-induced lipotoxic heart disease.

## Introduction

Obesity is associated with increased risk of cardiac failure and death [1], [2]. As a consequence of the increasing prevalence of obesity it is pertinent to improve understanding of the metabolic alterations causing cardiac dysfunction in overweight individuals. Obesity instigates intracellular accumulation of triglycerides in cardiac myocytes [3]–[5]. Lipid accumulation and altered metabolism of free fatty acids are associated with development of myocardial contractile dysfunction and may lead to cardiac myocyte apoptosis [5]–[8]. Although, the mechanisms involved in development of obesity-associated heart disease remain to be fully clarified, they appear to be independent from those leading to ischemia-induced heart dysfunction. Consequently, lipotoxic heart disease has been proposed as a distinct form of cardiomyopathy associated with obesity and type 2 diabetes [9]–[11].

Lipid accumulation in the heart results from an imbalance between uptake and utilization of free fatty acids. Under normal conditions, ∼70% of the energy production in the heart is derived from fatty acids [10]. Heart failure is accompanied by myocyte lipid accumulation, which has been explained by a switch in substrate utilization from fatty acids to glucose [12] leading to storage of the excess fatty acids as triglycerides. However, obesity is accompanied by both increased utilization of fatty acids for energy production and lipid accumulation both in humans and mice [4], [5], [13]. This apparent paradox may be explained by increased delivery of fatty acids to the heart in obesity [14], [15]. Increased plasma free fatty acids promote increased cardiac uptake reflecting the concentration gradient between plasma and the intracellular milieu in the cardiac myocytes [16], [17]. Moreover, obesity and cardiac lipid accumulation has been associated with increased heart expression of genes that stimulate local production (*i.e.* lipoprotein lipase (LPL)) and transport protein mediated uptake (*i.e.*, fatty acid translocase (FAT/CD36), fatty acid transporter protein 1 (FATP1) and fatty acid transporter protein 4 (FATP4)) of free fatty acids [5]. The regulatory role of cardiac free fatty acid uptake in lipotoxic heart disease is underscored by seminal studies showing that overexpression of LPL and FATP1 causes cardiac triglyceride accumulation and cardiac dysfunction in lean mice [18], [19]. In the obese heart, the increased availability of free fatty acids is also associated with upregulation of genes involved with fatty acid metabolization and increased utilization of fat for energy production [8].

Divergence of the net metabolic flux of fatty acids from the oxidative pathway leads to myocardial triglyceride accumulation and development of lipotoxic heart disease in mouse models that overexpress either PPARα or long-chain acyl-CoA synthetase (ACSL) [20], [21], or are long-chain acyl-CoA dehydrogenase-deficient (LCAD) [22]. The obese rat heart displays increased expression of uncoupling protein 3 (UCP3) shunting free fatty acids out of the mitochondria and increasing cytosolic fatty acid levels [23]. The excess availability of fatty acids that are not used for energy production leads to accumulation of intracellular triglycerides. The turnover time of triglycerides in the heart is extremely rapid (∼5 hours) compared to adipose tissue (200–270 days) [10], [24], [25]. This implies that cardiac triglyceride accumulation is not inert but rather is associated with markedly increased intracellular fluxes of free fatty acids.

The human heart expresses the two genes that are mandatory for formation of apoB-containing lipoproteins, i.e. the microsomal triglyceride transfer protein (MTP) gene and the apoB gene [26]. MTP is an intracellular protein located in the endoplasmatic reticulum, which transfers neutral lipids onto the apoB polypeptide and participates in the subsequent lipidation of the nascent lipoproteins [27]. Two recent reports independently demonstrated that there are two isoforms of the MTP gene in the mouse MTP-A and MTP-B (also named MTP and MTPv1) [28], [29]. The MTP-B variant has a unique exon 1 located 2.7 kb 5′ to the canonical exon 1 resulting in translation of a different signal peptide and addition of three amino acids at the NH2-terminus of the mature MTP protein [28], [29]. The physiological role of apoB is to package and secrete lipids in the form of lipoproteins. Indeed, cardiac myocytes secrete apoB-containing lipoproteins [30] and cardiac triglyceride accumulation is attenuated in apoB-overexpressing transgenic mice with streptozotocin-induced type 1 diabetes [31], lipoprotein lipase overexpressing mice [32], and LCAD-deficient mice [33]. These findings indicate that export of triglycerides in the form of lipoproteins can reduce pathological triglyceride accumulation. Therefore, export of triglycerides in the form of lipoproteins may represent a novel pathway in cardiac lipid metabolism that deserves further exploration.

We have utilized mice overexpressing a full length human apoB transgene and mice lacking cardiac expression of MTP-A to explore the role of cardiac lipoprotein formation on lipid metabolism and heart function in mice with diet-induced obesity. The data suggest that local lipoprotein secretion is integrated with cardiac lipid metabolism and protects against obesity-induced lipotoxic heart disease.

## Results

### Induction of MTP-B in MTP-A-deficient mouse hearts

*Ob/ob* mice have increased cardiac triglyceride stores, which are associated with increased total MTP mRNA expression and MTP activity in the myocardium [5]. This may reflect that increased lipoprotein formation is a compensatory mechanism dampening cardiac lipid accumulation in the setting of excess supplies of fatty acids.

**Figure 1:**
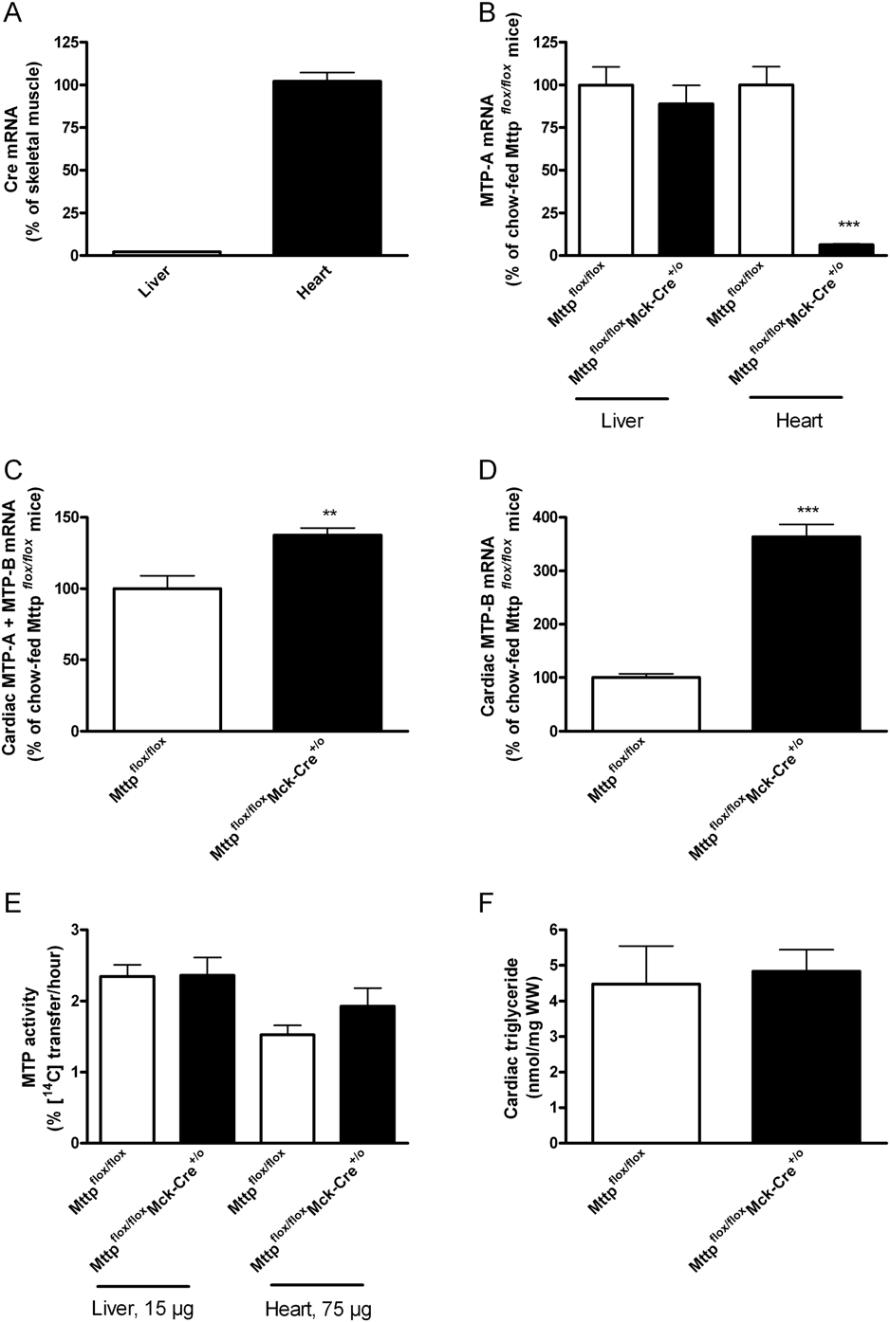
Effect of heart-specific MTP-A-deficiency in chow-fed mice. Mttp^flox/flox^ mice were bred with Mck-Cre^+/o^ mice to generate mice with heart-specific MTP-A-deficiency. A) Cre mRNA expression in liver (*n* = 4) and heart (*n* = 4) of Mttp^flox/flox^Mck-Cre^+/o^ mice, B) MTP-A mRNA expression in the liver (*n* = 4 in each group) and heart (*n* = 8 in each group) of Mttp^flox/flox^ and Mttp^flox/flox^Mck-Cre^+/o^ mice, C) Total MTP mRNA expression in the heart of Mttp^flox/flox^ (*n* = 8) and Mttp^flox/flox^Mck-Cre^+/o^ mice (*n* = 8), D) Cardiac MTP-B mRNA expression in Mttp^flox/flox^ (*n* = 8) and Mttp^flox/flox^Mck-Cre^+/o^ mice (*n* = 8), E) Cardiac MTP activity Mttp^flox/flox^ (*n* = 6) and Mttp^flox/flox^Mck-Cre^+/o^ mice (*n* = 6), F) Cardiac triglycerides in Mttp^flox/flox^ (*n* = 8) and Mttp^flox/flox^Mck-Cre^+/o^ mice (*n* = 8). *Open bars*: Mttp^flox/flox^ mice and *closed bars*: Mttp^flox/flox^Mck-Cre^+/o^ mice. Values are mean±SEM. The *p* values for two-group comparisons are: ** *P*<0.01; *** *P*<0.005 compared to chow-fed controls.

Real-time PCR studies with MTP-A and MTP-B specific assays showed that the expression of both isoforms is increased in mice with diet-induced obesity after fat-feeding (60% energy from fat) for ∼1 yr (Fig. S1). To explore the importance of the two MTP isoforms in the heart of obese mice, we bred mice with loxP sites flanking exon 1 of the MTP-A gene (Mttp^flox/flox^) [34] with transgenic mice that express Cre-recombinase in the heart and skeletal muscle, but not in liver (Mck-Cre^+/o^ mice) (Fig. 1A). Predictably, deletion of exon-1 of MTP-A should not abolish MTP-B expression since exon 1 of the MTP-B isoform is located ∼2.7 kb upstream of exon 1 of MTP-A. Chow-fed (12% energy from fat) Mttp^flox/flox^Mck-Cre^+/o^ mice had a >95% reduction of MTP-A mRNA in the heart and unchanged MTP-A mRNA expression in the liver as compared with Mttp^flox/flox^ mice indicating that ablation of MTP-A in cardiac myocytes was highly effective and tissue specific (Fig. 1B). Nevertheless, the cardiac total MTP (MTP-A+MTP-B) mRNA expression was increased 1.4 fold in Mttp^flox/flox^Mck-Cre^+/o^ mice compared with Mttp^flox/flox^ littermates (Fig. 1C). This increase was attributable to a ∼3.6 fold elevation (*P* = 0.0006) of the MTP-B mRNA expression (Fig. 1D) and may reflect increased transcription of MTP-B from the Cre-loxP-modified MTP gene locus.

The ability of the MTP-B isoform to mediate triglyceride transfer was evaluated by incubating cardiac microsomal protein fractions from Mttp^flox/flox^Mck-Cre^+/o^ mice with ^14^C-trioleate-labeled lipid vesicles. Cardiac MTP activity was similar in Mttp^flox/flox^Mck-Cre^+/o^ mice and with Mttp^flox/flox^ littermates (Fig. 1E). Moreover, cardiac triglyceride and cholesterol concentrations were similar in chow-fed, non-fasted Mttp^flox/flox^Mck-Cre^+/o^ mice and Mttp^flox/flox^ littermates (Fig. 1F and data not shown). These results indicate that upregulation of MTP-B can compensate for the loss of MTP-A in the heart of lean chow-fed mice.

### Effect of MTP-A deficiency on lipid accumulation and expression of lipid metabolizing genes in obese mouse heart

**Figure 2:**
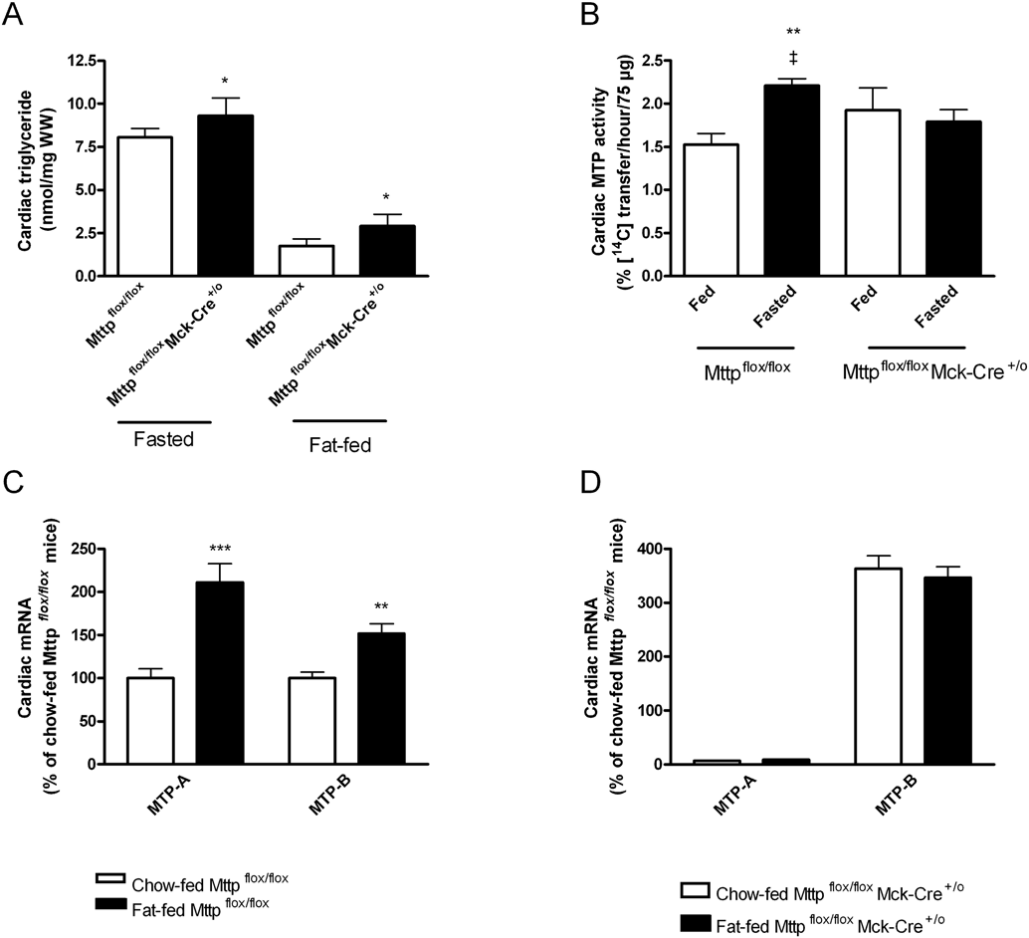
Effect of heart-specific MTP-A deficiency on cardiac lipid accumulation and MTP activity in fasted mice and fat-fed obese mice. A) cardiac triglycerides in mice that were fasted for 18 hours and mice that were fat-fed for three months (*n* = 7–9 in each group). *P<0.05 compared to Mttp^flox/flox^ mice. B) MTP activity in fasted versus chow-fed mice (*n* = 6 in each group). **P<0.01 compared to fed mice, ‡ *P*<0.01 fasted Mttp^flox/flox^ compared to fasted Mttp^flox/flox^Mck-Cre^+/o^ mice A+B) *Open bars*: Mttp^flox/flox^ mice and *closed bars*: Mttp^flox/flox^Mck-Cre^+/o^ mice. C) MTP-A and MTP-B mRNA expression in fat-fed (*closed bars*) versus chow-fed (*open bars*) Mttp^flox/flox^ mice (*n* = 7 in each group). **P<0.01 and ***P<0.005 compared to chow-fed Mttp^flox/flox^ mice. D) MTP-A and MTP-B mRNA expression in fat-fed (*closed bars*) versus chow-fed (*open bars*) Mttp^flox/flox^Mck-Cre^+/o^ mice (*n* = 7 in each group). Values are mean±SEM.

Previous studies suggest that mice with heart-specific MTP-A deficiency accumulate excess cardiac triglycerides after fasting [33]. Fasting increases the delivery of free fatty acids to the heart and causes cardiac triglyceride accumulation [16]. Thus, it is conceivable that the effect of MTP-A deficiency only becomes evident in the setting of surplus supplies of fatty acids and as such could be important in obese mice. We examined this possibility by comparing hearts from Mttp^flox/flox^Mck-Cre^+/o^ mice and Mttp^flox/flox^ littermates that had been fed a chow diet and fasted for 18 hours or had been fed a fat-enriched diet for 3 months. After fasting or fat-feeding the cardiac triglyceride content was higher in Mttp^flox/flox^Mck-Cre^+/o^ mice compared with Mttp^flox/flox^ littermates (*P*<0.05) (Fig. 2A). Notably, after fasting the cardiac MTP activity was increased in Mttp^flox/flox^ mice (*P* = 0.009) but not in Mttp^flox/flox^Mck-Cre^+/o^ mice (Fig. 2B). Moreover, after fat feeding MTP-A and MTP-B expression increased in Mttp^flox/flox^ control mice (Fig. 2C) whereas MTP-B expression was unaffected in Mttp^flox/flox^Mck-Cre^+/o^ mice (Fig. 2D). The present data thus imply that the excess cardiac triglyceride accumulation in Mttp^flox/flox^Mck-Cre^+/o^ mice occurs due to defect transcriptional activation of the ablated MTP gene locus after fasting- or fat-feeding. Moreover, the results thus support the notion that a compensatory increase in cardiac MTP expression protects against cardiac triglyceride accumulation in the obese mouse heart.

**Figure 3:**
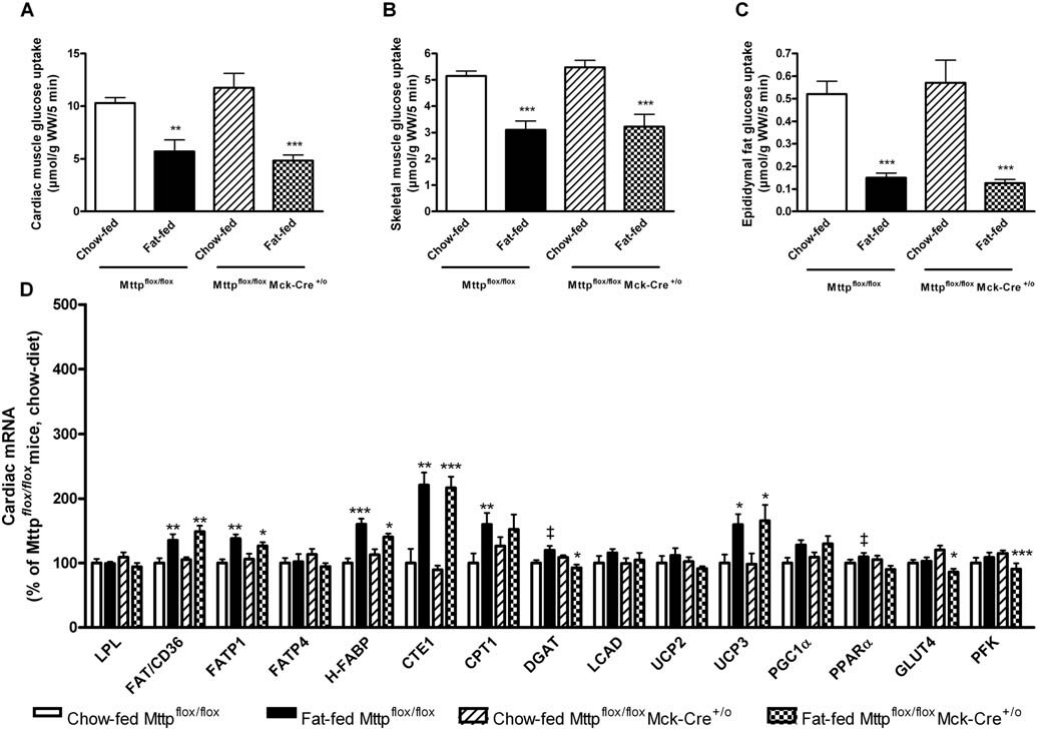
Effect of heart-specific MTP-A deficiency on insulin-stimulated glucose uptake and expression of lipid metabolizing genes in obese mouse heart. The effect of heart-specific MTP-A deletion on A) insulin-stimulated glucose uptake in cardiac ventricles, B) skeletal muscle, and C) epididymal fat was determined after 3 months of fat-feeding. D) Cardiac mRNA expression was quantified with real-time PCR in fat-fed male Mttp^flox/flox^ mice, Mttp^flox/flox^Mck-Cre^+/o^ mice and their lean controls. Values are after 3 months of diet. *Open bars*: chow-fed Mttp^flox/flox^ mice (n = 7–8), *closed bars*: fat-fed Mttp^flox/flox^ mice (n = 7–8), *hatched bars*: chow-fed Mttp^flox/flox^Mck-Cre^+/o^ mice (n = 7–8), *squared bars*: fat-fed Mttp^flox/flox^Mck-Cre^+/o^ mice (n = 7). Values are mean±SEM. The *p* values for two-group comparisons are: * *P*<0.05, ** *P*<0.01; *** *P*<0.005 compared to chow-fed controls; ‡ *P*<0.01 compared to fat-fed Mttp^flox/flox^Mck-Cre^+/o^ mice.

Diet-induced obesity has been associated with impaired insulin-stimulated glucose uptake in skeletal muscle [35] and altered lipid metabolism in cardiac muscle [20]. There was no excess triglyceride accumulation in the hearts of Mttp^flox/flox^ control mice after 3 months of fat-feeding (compare Fig. 1F and Fig 2A) which is in accord with previous findings by Somoza *et al* [13]. Nevertheless, insulin-stimulated glucose uptake was markedly reduced in the heart (as well as in skeletal muscle and adipose tissue) when insulin was injected intravenously together with 2-deoxy-[^3^H]glucose 25 minutes prior to removal of the mouse heart (Fig. 3A–C). The decrease in cardiac insulin-sensitivity occurred without changes in cardiac glucose transporter-4 (GLUT4) and (PFK) mRNA expression (involved in uptake and oxidation of glucose) (Fig 3D). Fat-feeding of the Mttp^flox/flox^ control mice for 3 months also caused significant increases in the expression of several lipid metabolizing genes which mediate free fatty acid uptake, *i.e.* FAT/CD36, FATP1, FATP4, intracellular transport, *i.e.* heart-fatty acid binding protein (H-FABP), removal of the CoA group from acyl-CoA in the cytosol, *i.e.* cytosolic acyl-CoA thioesterase 1 (CTE1), mitochondrial uptake of fatty acids, *i.e.* carnitine palmitoyltransferase 1b (CPT1b), and shunting of fatty acids out the mitochondria, *i.e.* UCP3 (Fig. 3D). This suggests that diet-induced obesity causes alterations in cardiac glucose and lipid metabolism that precedes lipid accumulation in myocytes.

There was no difference in cardiac insulin-stimulated glucose uptake between Mttp^flox/flox^Mck-Cre^+/o^ and Mttp^flox/flox^ control mice (Fig 3A). However, the mRNA expression of diacylglycerol acyltransferase (DGAT) and PPARα was reduced in fat-fed Mttp^flox/flox^Mck-Cre^+/o^ mice compared with the fat-fed Mttp^flox/flox^ mice (Fig. 3D), supporting the notion that rates of lipoprotein secretion may modulate cardiac triglyceride homeostasis in the setting of diet-induced obesity.

### Cardiac triglyceride accumulation in fat-fed obese mice is prevented by overexpression of human apoB in the heart

**Figure 4:**
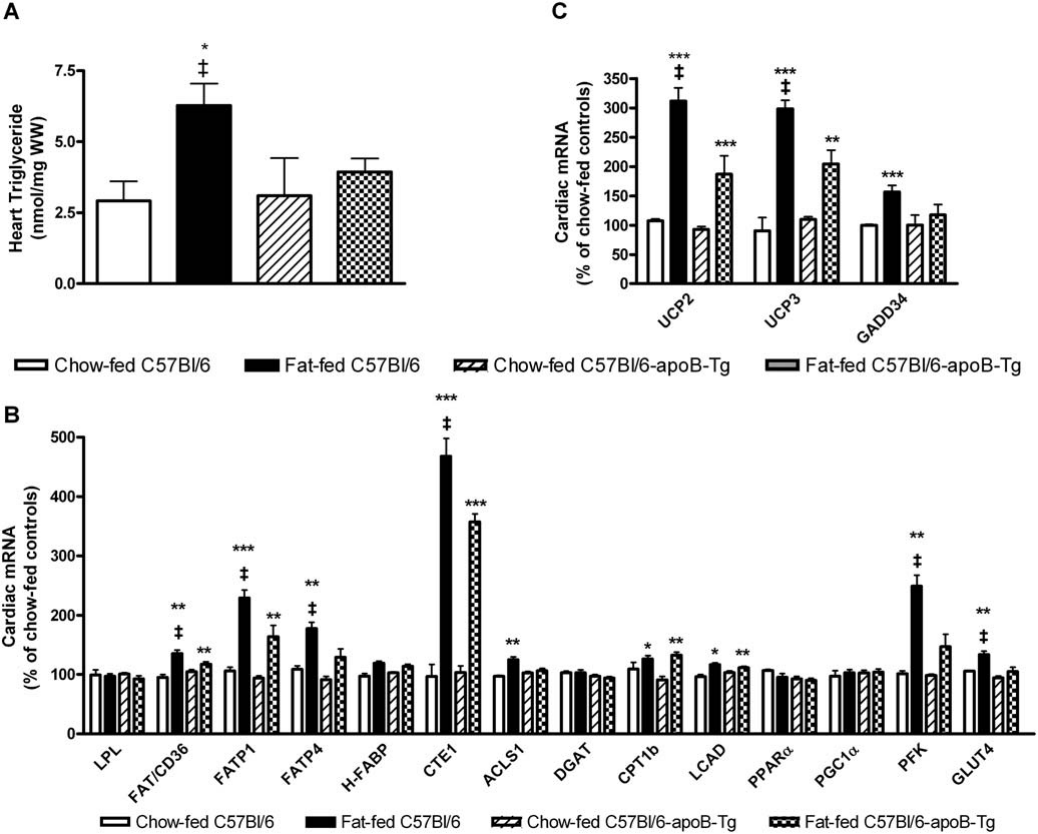
Overexpression of human apoB in the heart affects cardiac triglyceride levels as well as expression of genes involved in cardiac stress and metabolism of free fatty acids in fat-fed C57Bl/6 mice. The effect of cardiac overexpression of a human apoB-transgene in obese mice on A) cardiac triglycerides were determined using TLC, B) cardiac mRNA expression of lipid metabolising genes and C) cardiac mRNA expression of stress-related genes was quantified with real-time PCR in male fat-fed C57Bl/6 (*n* = 11) and C57Bl/6-apoB-Tg mice (*n* = 9) and their lean controls (*n* = 6). Values are after 12 months of diet. *Open bars*: chow-fed C57Bl/6 mice, *closed bars*: fat-fed C57Bl/6 mice, *hatched bars*: chow-fed C57Bl/6-apoB-Tg mice, *squared bars*: fat-fed C57Bl/6-apoB-Tg mice. Values are mean±SEM. The *p* values for two-group comparisons are: * *P*<0.05, ** *P*<0.01; *** *P*<0.005 compared to chow-fed controls; ‡ *P*<0.05 Fat-fed C57Bl/6 compared to fat-fed C57Bl/6-apoB-Tg.

Prolonged excess caloric intake increases cardiac triglyceride stores in mice [5]. We fed the fat-enriched diet to human apoB-transgenic male mice and litter-mate C57Bl/6 controls for ∼1 year to explore the impact of increased rates of cardiac lipoprotein formation in the setting of obesity and increased cardiac triglyceride stores. Fat-feeding induced equal increases in body weight, plasma insulin, leptin, and glucose in male C57Bl/6 mice and their apoB-transgenic littermates (Fig. S2A–F). Fasting plasma concentrations of free fatty acids after 9 months of fat-feeding were increased in to a similar extent in fat-fed C57Bl/6 mice and human apoB-transgenic littermates (0.80±0.03 mmol/L and 0.87±0.05 mmol/L) compared with chow-fed control mice (0.66±0.07 mmol/L and 0.34±0.01 mmol/L) (*P*<0.0001). After 12 months of fat-feeding, the heart triglyceride content was 110% increased in obese C57Bl/6 mice compared with lean C57Bl/6 mice (Fig. 4A). The obesity-induced increase in cardiac triglycerides was abolished in human apoB-transgenic mice (Fig. 4A). Neither cardiac cholesterol (data not shown) nor ceramide levels were affected by fat-feeding or overexpression of the human apoB transgene. The cardiac ceramide concentration was 20±1 nmol/mg wet weight (ww) in fat-fed versus 18±1 nmol/mg ww in chow-fed C57Bl/6 mice (*P* = 0.15), and 21±2 nmol/mg ww in fat-fed versus 23±2 nmol/mg ww in chow-fed apoB-transgenic mice (*P* = 0.61). These results suggest that increased lipoprotein formation in cardiac myocytes prevents triglyceride accumulation without affecting cholesterol or ceramide stores in hearts of long-term fat-fed obese mice.

### Overexpression of human apoB in the heart affects expression of genes involved in cardiac metabolism of free fatty acids

To judge whether increased lipoprotein formation affect metabolism of free fatty acids in hearts with excess lipid accumulation, we quantified the expression of selected genes controlling key steps in cardiac fatty acid metabolism. The mRNA levels of FAT/CD36, FATP1 and FATP4, CTE1, ACSL1 which couples the CoA group to fatty acids in the cytosol, CPT1b and LCAD which is involved in β-oxidation of fatty acids, were all increased in hearts from 1-yr fat-fed C57Bl/6 mice compared to chow-fed mice respectively (Fig. 4B). Notably, the magnitudes of the changes in gene expression were much more pronounced after fat-feeding for 1 year than after 3 months (compare Fig 3D and 4B). In contrast to the findings in C57Bl/6 mice, the cardiac expression of genes associated with fatty acid metabolizing showed less or no increase in fat-fed human apoB-transgenic mice (Fig. 4B). Thus, the expression levels of FAT/CD36, FATP1, FATP4, CTE1, and ACLS1 were significantly lower in hearts from fat-fed human apoB-transgenic than in the fat-fed C57Bl/6 littermates (Fig. 4B). These results suggest that increased lipoprotein formation attenuates changes in gene expression that increase uptake and metabolization of free fatty acids in the hearts of long-term fat-fed mice.

In accordance with previous results in obese *ob/ob* mice [5] and *fa/fa* Zucker rats [36] GLUT4 as well as PFK displayed increased mRNA expression in the fat-fed C57Bl/6 mouse hearts. In hearts from fat-fed versus chow-fed human apoB-transgenic mice we did not identify this difference in GLUT4 and PFK mRNA expression (Fig. 4B). This suggests that normalization of cardiac lipid stores not only affects lipid metabolism but also prevents changes in expression of essential genes governing glucose metabolism.

### Overexpression of human apoB in the heart attenuates deterioration of cardiac function in fat-fed obese mice

**Figure 5:**
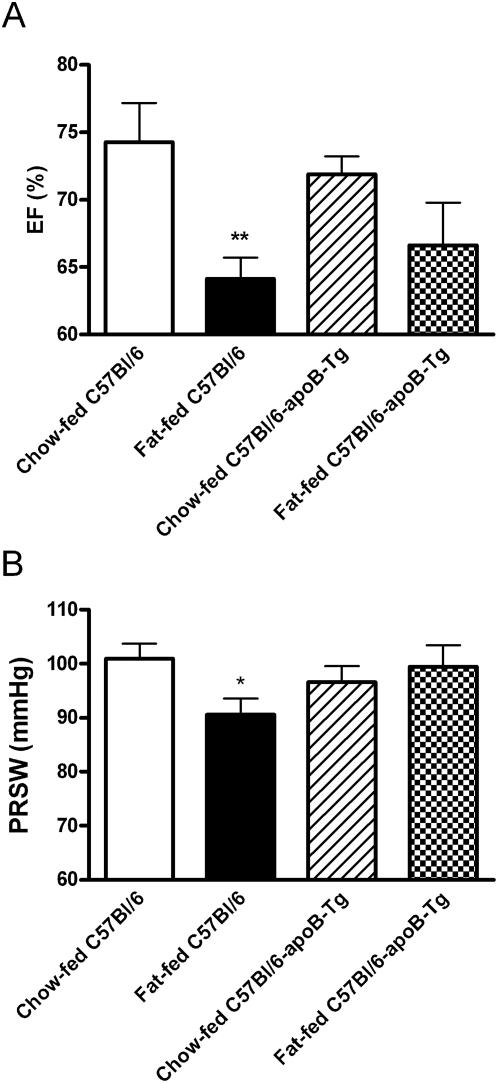
Overexpression of human apoB in the heart attenuates deterioration of cardiac function in fat-fed obese mice. Heart function was determined with a conductance catheter placed in the left cardiac ventricle A) EF, ejection fraction, B) PRSW, preload recruitable stroke work. *Open bars*: chow-fed C57Bl/6 mice (*n* = 7), *closed bars*: fat-fed C57Bl/6 mice (*n* = 7), *hatched bars*: chow-fed C57Bl/6-apoB-Tg mice (*n* = 11), *squared bars*: fat-fed C57Bl/6-apoB-Tg mice (*n* = 7). Values are mean±SEM after 11 months on the diets. The *p* values for two-group comparisons are: * *P*<0.05, ** *P*≤0.01.

Recent data demonstrated that fat-feeding of C57Bl/6 male mice results in deterioration of heart function [35]. This may result from cardiac lipid accumulation and increased uptake and metabolization of free fatty acids [37]. We therefore hypothesized that mitigated cardiac triglyceride accumulation and dampened uptake and metabolization of fatty acids in human apoB transgenic mice might alleviate adverse effects of fat-feeding on heart function. To test this idea, we examined *in vivo* heart function in human apoB-transgenic male mice and littermate C57Bl/6 controls that had been fat- or chow-fed for 11 months. Ejection fraction (EF) and the load-independent index, preload recruitable stroke work (PRSW) were decreased in fat-fed C57Bl/6 mice compared with chow-fed C57Bl/6 mice (Fig. 5A and 5B). In addition, the heart rate was reduced in the fat-fed versus chow-fed C57Bl/6 mice (Supplementary Table S1). This was in contrast to human apoB transgenic mice where neither of these variables was significantly altered in fat-fed compared to chow-fed mice (Fig. 5A and 5B and Supplementary Table S1). We did not observe any effects of fat-feeding or apoB overexpression on electrocardiographic recordings (data not shown). The results are compatible with the idea that increased lipoprotein formation in cardiac myocytes attenuates development of cardiac dysfunction in fat-fed obese mice.

Obesity and type 2 diabetes increase intracellular stress promoting apoptosis and insulin resistance [38], [39]. Increased expression of uncoupling protein 2 (UCP2) and UCP3 (directing fatty acids fluxes away from oxidative pathways) and of the pro-apoptotic growth arrest and DNA-damage-inducible gene 34 (GADD34) mRNA are markers of myocardial stress. For all three genes, the expression was increased in the heart of long-term fat-fed versus chow-fed C57Bl/6 mice, but was less or unaffected in fat-fed versus chow-fed human apoB-transgenic mice (Fig. 4C). GADD34 expression can be induced by a number of pathways including stress in the endoplasmatic reticulum [40]. However, cardiac mRNA expression of two markers of ER stress (immunoglobulin heavy chain-binding protein (BiP) and C/EBP-homologous protein (CHOP)) was not affected by fat feeding (data not shown).

## Discussion

The present data suggest that cardiac secretion of apoB-containing lipoproteins plays an integrated role in cardiac fatty acid metabolism and affects myocardial function in obese mice. Failure to upregulate MTP activity in fasted and short-term fat-fed mice with targeted MTP-A expression increased cardiac triglyceride stores. Overexpression of apoB attenuated triglyceride accumulation in the heart of long-term fat-fed obese mice. Thus, genes controlling lipoprotein synthesis, in addition to genes controlling rates of uptake and utilization of fatty acids in energy production, are significant determinants of cardiac triglyceride homeostasis in the obese heart.

Expression of MTP is mandatory for lipoprotein formation [41]. Both the canonical MTP-A isoform and the recently discovered MTP-B isoform are expressed in the murine heart. The expression of both MTP isoforms is increased in the heart of fat-fed obese mice suggesting that MTP is integrated in cardiac lipid metabolism. Complete MTP deficiency is embryonically lethal in mice [42]. We used mice with heart-specific deficiency of the MTP-A gene to study the role of MTP in the heart. Based on the comparison of the expression of total MTP and MTP-B mRNA in wild-type and MTP-A deficient mice, it can be estimated that ∼35% of the total amount of MTP mRNA represents the MTP-B transcript and ∼65% represents the MTP-A transcript in the mouse heart. The MTP-B isoform likely is functionally active in cardiac lipoprotein formation since the MTP-A deficient mouse hearts expressed the same MTP activity as control hearts and did not accumulate triglycerides when the mice were on a normal chow. Notably, direct evidence of MTP-B mediated lipoprotein formation in the heart was not provided by the present studies. Nevertheless, it is interesting that mice with heart-specific MTP-A deficiency, in contrast to wild type mice, could not upregulate MTP expression and accumulated excess triglycerides in the heart in response to fasting or 3 months of fat-feeding. This suggests that upregulation of cardiac MTP expression serves to protect against myocardial lipid accumulation when the supply of fatty acids exceeds the need for energy production. A promoter polymorphism in the MTP gene is associated with decreased MTP gene expression and excess cardiovascular mortality in patients with ischemic heart disease [43]. The present data thus warrants further studies to determine whether MTP promoter polymorphisms could also affect the risk of obesity-induced heart disease.

Triglyceride accumulation in skeletal muscle is associated with insulin resistance [44]. Recent studies, however, suggest that increased muscle uptake and metabolism of free fatty acids rather than fat accumulation in itself causes the insulin resistance in obesity [45], [46]. The present findings in the mouse heart are in accordance with this idea. Thus, the heart was insulin resistant in obese mice after 12 weeks of fat feeding. At this time point there was increased cardiac expression of lipid metabolizing genes but no excess cardiac triglyceride accumulation (cardiac triglycerides in Mttp^flox/flox^ control mice were actually lower in the fat-fed group than in the chow-fed group, as seen in Fig. 1F and Fig. 2A). Moreover, cardiac insulin sensitivity was unaffected by the increased cardiac triglyceride stores in Mttp^flox/flox^Mck-Cre^+/o^ mice.

Several studies demonstrates that obesity-induced changes in cardiac energy metabolism involve increased uptake and utilization of free fatty acids that ultimatively lead to cardiac lipid accumulation [5], [47], [48]. Altered fatty acid metabolism leads to decreased metabolic flexibility which is detrimental to the function of the myocardium, *e.g.* due to increased formation of reactive oxygen species [49]–[51]. Interestingly, overexpression of apoB in the heart of long-term-fat-fed obese mice not only prevented cardiac triglyceride accumulation but also reduced the effect of fat-feeding and obesity on genes controlling fatty acid metabolism in the heart. The reduction of cardiac triglycerides in the fat-fed apoB transgenic mice was associated with partial normalization of the expression of genes that are key regulators of cardiac fatty acid uptake, cytosolic release of free fatty acids from acyl-CoA, and uncoupling of fatty acid β-oxidation in mitochondria. Increased expression of FAT/CD36, FATP1, UCP2 and UCP3 have all been implicated in development of lipotoxic heart disease [20], [52], [53]. Thus, the results are compatible with the idea that normalization of cardiac lipids via increased export in lipoproteins leads to decreased uptake and metabolism of fatty acids and confers at least partial resistance to the detrimental effect of obesity on myocardial function. Notably, normalization of triglyceride store also reduced the expression of genes involved with glucose metabolism and the GADD34 marker of cardiac stress. Thus, the data suggest that reduction of cardiac triglyceride stores have effects on cardiac energy utilization that exceeds lipid metabolism and, as such, is a key controlling factor in precipitation of lipotoxic heart disease in obesity. It should be noted, however, that this conclusion is based on mRNA expression data and further studies are needed to explore the impact of lipoprotein formation on cardiac energy metabolism *in vivo*.

In conclusion, the present results support that cardiac lipoprotein synthesis and secretion is important for controlling the triglyceride storage in the heart of mice when fatty acid supplies are increased, such as in obesity. In addition, the export of triglycerides in lipoproteins appears to reduce the effect of obesity on the otherwise perturbed expression profile of free fatty acid metabolizing genes and protect against adverse effects of obesity on myocardial function.

## Materials and Methods

### Mice

Mice with heart-specific MTP-A deficiency were obtained by breeding MTP loxp mice (stock no: 003902, Jackson Laboratories, USA) with Mck-Cre mice [54] to obtain Mttp^flox/flox^Mck-Cre^+/o^ mice and Mttp^flox/flox^ littermate controls. For the study of cardiac MTP-activity and response to fasting the mice were kept on the standard chow diet. For the study of obesity-associated effects on the heart, 7-week-old mice were randomly assigned to either the high-fat diet or the standard chow diet for 12 weeks. At the end of the fat-feeding period the body weight was 43.4±1.7 g (*n* = 7) in fat-fed Mttp^flox/flox^, 45.5±1.8 g (*n* = 8) in Mttp^flox/flox^Mck-Cre^+/o^, 31.2±0.6 g (*n* = 8) in chow-fed Mttp^flox/flox^, and 30.6±1.0 g (*n* = 8) in chow fed Mttp^flox/flox^Mck-Cre^+/o^ mice. Seven-week-old male C57Bl/6 and human apoB-transgenic mice (B6.SJL-Tg(APOB)1102Sgy-mice backcrossed to the C57Bl/6 background for >20 generations) littermates were randomized to a high-fat diet with 60% fat (D12492, Research Diets) or a standard chow diet with 12% fat (Altromin 1314, Brogaarden, Denmark). Heart function was studied after 45 weeks on the diet whereas cardiac gene expression and lipid accumulation was studied in separate mice after 52 weeks on the diet. The mice fed for 52 weeks were also used as a part of another study of atherosclerosis [55]. The animals were housed under temperature-controlled conditions with free access to food and water. The studies were approved by the Danish Animal Experiments Inspectorate (Dyreforsoegstilsynet).

### Blood and tissue samples

Blood were drawn in pre-cooled tubes containing Na_2_-EDTA and centrifuged at 4000·*g* for 10 minutes at 4°C. The hearts were carefully cleaned from pericardial fat and atrial tissue before snap frozen in liquid nitrogen. Tissue and plasma samples were stored at −80°C until use.

### Plasma biochemistry

Plasma glucose in tail blood was determined with a Medisense PC*x* glucose meter (Abbott Laboratories A/S, Gentofte, Denmark). Plasma free fatty acids concentrations were determined with an enzymatic kit (Wako NEFA C kit, TriChem Aps, Frederikssund, Denmark). Sandwich elisa assays were used to measure plasma insulin and leptin (catalogue no. EIA-3440, DRG, Germany, and catalogue no. RD291001200R, BioVendor, Heidelberg, Germany, respectively).

### Cardiac triglycerides

Lipids were extracted, re-dissolved in toluene, and separated with TLC prior to quantification as previously described [56].

### Cardiac ceramides

Cardiac ceramides were purified from lipid extracts after addition of C17-ceramide (Avanti Polar Lipids, Alabaster, AL, US) as internal standard. Ceramides were extracted by solid phase extraction (Strata NH2, Part Number: 8B-S009-HBJ), hydrolyzed to free sphingosine which was derivatized with O-phtalaldehyde (Sigma, Brøndby, Denmark) before separated on a C18 HPLC column (Gemini 5 µm C18 110 Å Column 250×3.0 mm) (Phenomenex, Allerød, Denmark) and detected as fluorescence at 450 nm (excitation at 340 nm).

### Cardiac gene expression

Gene expression was measured with real-time PCR. mRNA was extracted using the Trizol reagent (Invitrogen, Taastrup, Denmark), quantified spectrophotometricly, and the quality was assessed with capillary electrophoresis (2100 bioanalyzer, Agilent Technologies). cDNA was made with M-MULV (Roche) and real-time PCR analyses were performed with SYBR-Green and the Light Cycler instrument (Roche). Primer sequences for mouse ACSL1, BiP, CHOP, CTE1, Cre, GADD34, MTP-A, MTP-B, MTP-A+MTP-B, UCP2, and UCP3 are shown in Supplementary Table S2. Sequences for human apoB, mouse apoB, CPT1b, DGAT, FAT/CD36, FATP1, FATP4, GLUT4, H-FABP, hypoxanthine-guanine phosphoribosyltransferase (HPRT), LCAD, LPL, PFK, PPARγ-coactivator-1α (PGC1α), and PPARα, have been published previously [5], [31], [57], [58]. The expression of the reference gene HPRT was used to normalize the expression of target genes.

### In vivo evaluation of heart function

The left ventricular function was evaluated by analysis of the pressure-volume loops acquired by conductance catheter technique [59]. The animals were intubated and mechanically ventilated with 2.2% isoflurane in 50% O_2_ and 50% room air at 100 breaths/min and a tidal volume of 10 µl/g. Temperature was maintained at 37°C by a rectal temperature-controlled heating pad and a heating lamp. An intravenous line was established through the left jugular vein and used for continuous infusion of isotonic NaCl at an infusion rate of 0.05 ml·g^−1^·h^−1^ throughout the protocol. The infusion of NaCl was discontinued for 1–2 min, and the ventilator was stopped at end expiration for 2–3 s before every measurement. The chest was entered via an anterior thoracotomy, and a 1.4-F four-electrode conductance catheter for mice (SPR-839; Millar Instruments, Houston, TX) was inserted into the left ventricle through the apex and positioned along the cardiac longitudinal axis. Pressure-volume loops were acquired with a signal-conditioning box (MPCU-200; Millar Instruments) using a 20-kHz excitation frequency and sampling rate of 1,000 Hz. The parallel conductance was estimated by intra venous injection of a 5 µL bolus of 30% hypertonic saline over approximately 0.5 s. The volume signal was calibrated by measuring the specific conductance of blood from each mouse by the cylinder method as previously described [60]. Maximal and minimal LV volumes and pressures during the cardiac cycle were used as end diastolic volume (EDV) and end systolic volume (ESV) and as end systolic pressure (ESP) and end diastolic pressure (EDP) respectively.

### MTP activity

MTP activity was assessed as previously described [5], [61]. Briefly, tissue biopsies of ∼50 mg were homogenised and the microsomal protein fraction was isolated by ultracentrifugation. Triglyceride transfer activity in the microsomal protein fraction was measured as transfer of ^14^C-trioleate-labeled lipid from donor vesicles to acceptor vesicle that contained only unlabeled triglycerides.

### Insulin stimulated glucose uptake in vivo

Fed mice were anesthetized with a mixture of fentanyl:droperidol:midazolam (0.02∶1.38∶0.14 mg/ml, 0.2 ml/10 g body weight), and a mixture of 0.005 IU insulin, 1.6 µCi of 2-deoxy-[^3^H]glucose and 1.0 µCi of [^14^C]sucrose/10 g body weight in saline with 0.1% BSA was administered in the a vein. [^14^C]Sucrose was used to calculate extracellular space. After 25 minutes blood was drawn in pre-cooled Na_2_EDTA tubes and heart, soleus muscle and the epididymal fat pad were isolated and rinsed carefully in ice-cold isotonic saline and snap-frozen in liquid nitrogen. Uptake of 2-deoxy-[^3^H]glucose in heart, soleus muscle, and epididymal fat was detected in perchloric acid extracts after corrected for label in the extracellular space as determined by the [^14^C] counts for sucrose.

### Statistics

Two-*g*roup comparison was performed with Student's *t-test* or non-parametric Mann-Whitney test when appropriate. *P*<0.05 was considered statistically significant. The effect of MTP-genotype on cardiac triglycerides was calculated with two-way ANOVA.

## Supporting Information

Figure S1 **MTP-A and MTP-B expression in the obese mouse heart.** Cardiac mRNA expression was quantified with real-time PCR in male fat-fed C57Bl/6 (*n* = 11) and and their lean controls (*n* = 6). Values are after 12 months of diet. *Open bars*: chow-fed C57Bl/6 mice, *closed bars*: fat-fed C57Bl/6 mice. Values are mean±SEM. The *p* values for two-group comparisons are: * *P*<0.05, *** *P*<0.005 compared to chow-fed controls.
(0.13 MB TIF)

Figure S2 **Effect of prolonged fat-feeding on basic metabolic parameters in male C57Bl/6 and C57Bl/6-apoB-Tg mice.** The effect of fat-feeding on A) bodyweight in C57Bl/6 mice, B) bodyweight in C57Bl/6-apoB-Tg mice, C) plasma glucose in C57Bl/6 and C57Bl/6-apoB-Tg mice, D) plasma insulin in C57Bl/6 and C57Bl/6-apoB-Tg mice, E) plasma leptin in C57Bl/6 and C57Bl/6-apoB-Tg mice. Values are after 11 months of diet and in overnight fasted mice. *Open bars*: chow-fed C57Bl/6 mice (*n* = 7), *closed bars*: fat-fed C57Bl/6 mice (*n* = 7), *hatched bars*: chow-fed C57Bl/6-apoB-Tg mice (*n* = 11), *squared bars*: fat-fed C57Bl/6-apoB-Tg mice (*n* = 7). Values are mean±SEM. The *p* values for two-group comparisons are indicated by: *** *P*<0.005 compared to chow-fed controls.
(0.22 MB TIF)

Table S1 **Heart function in fat-fed C57Bl/6 and C57Bl/6-apoB-Tg mice.** vData are from male mice chow- or fat-fed for 11 months. Values are mean±SEM, * *P*<0.05; ‡*P*<0.005 compared to lean controls. EDV indicates end-diastolic volume; ESV, end-systolic volume; ESP, end systolic pressure; EDP, end diastolic pressure; t, isovolumic relaxation time; EDVPR, end diastolic volume pressure relationship. *P*<0.05 is considered significant.
(0.03 MB DOC)

Table S2 **Primers used for real-time PCR.**
(0.04 MB DOC)
