# Supplementary material for: Cardiac Expression of Microsomal Triglyceride Transfer Protein Is Increased in Obesity and Serves to Attenuate Cardiac Triglyceride Accumulation
Source: PLoS One. 2009 Apr 23;4(4):e5300. doi: 10.1371/journal.pone.0005300 (PMC2668751; doi:10.1371/journal.pone.0005300)
Supplement: Table S1 — Heart function in fat-fed C57Bl/6 and C57Bl/6-apoB-Tg mice. vData are from male mice chow- or fat-fed for 11 months. Values are mean±SEM, * P<0.05; ‡P<0.005 compared to lean controls. EDV indicates end-diastolic volume; ESV, end-systolic volume; ESP, end systolic pressure; EDP, end diastolic pressure; t, isovolumic relaxation time; EDVPR, end diastolic volume pressure relationship. P<0.05 is considered significant. (0.03 MB DOC) [file pone.0005300.s003.doc]

Table S1. Heart function in fat-fed C57Bl/6 and C57Bl/6-apoB-Tg mice.

|  | C57Bl/6 | | C57Bl/6-apoB-Tg | |
| --- | --- | --- | --- | --- |
|  | Chow-fed | Fat-fed | Chow-fed | Fat-fed |
| Heart rate (beats/min) | 615 ± 8.6 | 586 ± 10.7* | 601 ± 6.9 | 588 ± 12.8 |
| EDV (µl) | 26.6 ± 1.7 | 30.4 ± 1.0 | 27.0 ± 2.0 | 32.0 ± 1.1 |
| ESV (µl) | 6.8 ± 0.88 | 10.9 ± 0.47‡ | 7.7 ± 0.82 | 10.5 ± 0.79 |
| SV (µl) | 19.8 ± 1.6 | 19.5 ± 1.0 | 19.3 ± 1.3 | 21.4 ± 1.5 |
| ESP (mmHg) | 98.1 ± 1.6 | 94.6 ± 4.9 | 97.1 ±1.3 | 106.7 ± 4.0* |
| EDP (mmHg) | 5.6 ± 0.4 | 6.8 ± 0.7 | 7.9 ± 1.0 | 7.3 ± 0.5 |
| τ (ms) | 5.0 ± 0.2 | 6.2 ± 0.6 | 5.7 ± 0.4 | 5.4 ± 0.1 |
| Diastolic stiffness (mmHg/ml) | 0.15 ± 0.02 | 0.16 ± 0.02 | 0.17 ± 0.04 | 0.18 ±0.04 |
| EDVPR (mmHg/µl) | 0.28 ± 0.06 | 0.20 ± 0.04 | 0.26 ± 0.04 | 0.19 ±0.03 |

Data are from male mice chow- or fat-fed for 11 months. Values are mean ± SEM, * *P*< 0.05; ‡ *P*< 0.005 compared to lean controls. EDV indicates end-diastolic volume; ESV, end-systolic volume; ESP, end systolic pressure; EDP, end diastolic pressure; τ, isovolumic relaxation time; EDVPR, end diastolic volume pressure relationship. *P*<0.05 is considered significant.
