# Supplementary material for: Cardiac Expression of Microsomal Triglyceride Transfer Protein Is Increased in Obesity and Serves to Attenuate Cardiac Triglyceride Accumulation
Source: PLoS One. 2009 Apr 23;4(4):e5300. doi: 10.1371/journal.pone.0005300 (PMC2668751; doi:10.1371/journal.pone.0005300)
Supplement: Table S2 — Primers used for real-time PCR. (0.04 MB DOC) [file pone.0005300.s004.doc]

**Table S2, Primers used for real-time PCR**

| Primer name | Primer sequence (from 5’-3’) | Reference |
| --- | --- | --- |
|  |  |  |
| ACLS1 5’ | CTGACAAGCCAGAAAAAGCC |  |
| ACLS1 3’ | TTTGGGGTTGCCTGTAGTTC |  |
|  |  |  |
| BiP 5’ | cagatcttctccacggcttc |  |
| BiP 3’ | gcggttttggtcattggtaa |  |
|  |  |  |
| CHOP 5’ | gcatgaaggagaaggagcag |  |
| CHOP 3’ | actgttcatgcttggtgcag |  |
|  |  |  |
| Cre 5’ | gcatttctggggattgctta |  |
| Cre 3’ | tgcatgatctccggtattga |  |
|  |  |  |
| CTE1 5’ | ctacgatgacctccccaaga |  |
| CTE1 3’ | ggagatggtgttcccaacag |  |
|  |  |  |
| GADD34 5’ | aggaccccgagattcctcta |  |
| GADD34 3’ | cctggaatcaggggtaaggt |  |
|  |  |  |
| MTP-A 5’ | atgatcctcttggcagtgcttt | [28] |
| MTP-A 3’ | attttgtagcccacgctgtc |  |
|  |  |  |
| MTP-B 5’ | acagtcgtgatggggaaatg |  |
| MTP-B 3’ | attttgtagcccacgctgtc |  |
|  |  |  |
| MTP-A+MTP-B 5’ | agaccagggcttttgccttg |  |
| MTP-A+MTP-B 3’ | gggaatggctttgtatttgg |  |
|  |  |  |
| UCP2 5’ | tctcctgaaagccaacctca |  |
| UCP2 3’ | ctacgttccaggatcccaag |  |
|  |  |  |
| UCP3 5’ | tgctgagatggtgacctacg |  |
| UCP3 3’ | tcagcatacagtgcagaggg |  |
|  |  |  |
